# Providing Adverse Outcome Pathways from the AOP-Wiki in a Semantic Web Format to Increase Usability and Accessibility of the Content

**DOI:** 10.1089/aivt.2021.0010

**Published:** 2022-03-17

**Authors:** Marvin Martens, Chris T. Evelo, Egon L. Willighagen

**Affiliations:** ^1^ Department of Bioinformatics—BiGCaT, NUTRIM, and Maastricht University, Maastricht, The Netherlands.; ^2^ Maastricht Centre for Systems Biology (MaCSBio), Maastricht University, Maastricht, The Netherlands.

**Keywords:** adverse outcome pathways, linked data, resource description framework, risk assessment, SPARQL

## Abstract

**Introduction:**

The AOP-Wiki is the main platform for the development and storage of adverse outcome pathways (AOPs). These AOPs describe mechanistic information about toxicodynamic processes and can be used to develop effective risk assessment strategies. However, it is challenging to automatically and systematically parse, filter, and use its contents. We explored solutions to better structure the AOP-Wiki content, and to link it with chemical and biological resources. Together, this allows more detailed exploration, which can be automated.

**Materials and Methods:**

We converted the complete AOP-Wiki content into resource description framework (RDF) triples. We used >20 ontologies for the semantic annotation of property–object relations, including the Chemical Information Ontology, Dublin Core, and the AOP Ontology.

**Results:**

The resulting RDF contains >122,000 triples describing 158 unique properties of >15,000 unique subjects. Furthermore, >3500 link-outs were added to 12 chemical databases, and >7500 link-outs to 4 gene and protein databases. The AOP-Wiki RDF has been made available at https://aopwiki.rdf.bigcat-bioinformatics.org

**Discussion:**

SPARQL queries can be used to answer biological and toxicological questions, such as listing measurement methods for all Key Events leading to an Adverse Outcome of interest. The full power that the use of this new resource provides becomes apparent when combining the content with external databases using federated queries.

**Conclusion:**

Overall, the AOP-Wiki RDF allows new ways to explore the rapidly growing AOP knowledge and makes the integration of this database in automated workflows possible, making the AOP-Wiki more FAIR.

## Introduction

Since its establishment in 2010, the adverse outcome pathway (AOP) concept has become a prominent tool for the risk assessment community.^1,2^ AOPs are a chain of biological processes, called Key Events (KEs), starting from a molecular perturbation with a stressor toward an Adverse Outcome (AO), connected by Key Event Relationships (KERs). AOPs exist to capture all mechanistic toxicological knowledge from literature and data, to direct future studies to fill gaps of existing knowledge, and to drive Integrated Approaches to Testing and Assessment (IATA) development.^1,3^ This was demonstrated with the AOP-based IATA for skin sensitization, resulting in various IATA with combinations of *in vitro* and *in silico* assays outperforming animal tests.^4^

The majority of the AOPs are developed and stored in the AOP-Wiki (https://aopwiki.org/), which is part of the AOP knowledge base, released in 2014 as a result of the AOP development program initiated by the Organisation for Economic and Collaborative Development.^5^ This wiki is designed to facilitate collaborative development of qualitative AOP descriptions, and thereby promote their incorporation into risk assessments and stimulate effective reuse of mechanistic toxicological knowledge.^6,7^

The resulting AOPs describe much of the biological context surrounding toxicological processes; most of the information on genes, chemicals, biological pathways, phenotypes, among other things, is already captured in specialized databases or ontologies outside of AOP-Wiki.^8^ However, the AOP-Wiki has limited possibilities for linking external information and data, mostly consisting of free-text descriptions and links to the U.S. CompTox Chemistry Dashboard^9^ and to NCBI for taxonomic applicability.^10^ An initiative to make the reporting more consistent was the introduction of KEs Components^11^ for the annotation of Biological Processes, Biological Objects, and Biological Actions for KEs, and annotations of cell types and organs in which KEs can occur.

Since the AOP-Wiki is the central repository for AOPs and therefore a key player in the shift toward animal-free testing strategies, it is essential that its contents can be queried and utilized effectively to answer biological questions and to reuse existing knowledge. However, accessing the data computationally or linking with other resources is hardly possible when only downloadable eXtensible Markup Language (XML) data dumps are provided that consist mostly of free text. Because of these aspects, parsing and querying the continuously growing amount of information in the AOP-Wiki is a complex, time-consuming task. This is a problem because it prevents the integration of AOP knowledge with other data and resources.

This could be resolved by applying Linked Open Data solutions, such as structuring the data in a resource description framework (RDF) model,^12^ introducing persistent identifiers and semantic annotations, and implementing Application Programming Interfaces (APIs) for accessing the data. RDF represents knowledge as semantic triples, in which a subject, predicate, and object together define a statement and assist in the meaningful representation of knowledge in a machine-readable manner.

These concepts are generally in line with the FAIR principles^13^ for data and knowledge management, developed to enhance the Findability, Accessibility, Interoperability, and Reusability of data and allow computational support of data usage; for example, the solutions applied by the Swiss Institute of Bioinformatics with the development of neXtProt Linked Data by implementing RDF annotations for easier exploration and retrieval of data through web services.^14,15^

Also, the use of ontologies and vocabularies for semantic annotations allows for the integration of data between resources, such as the direct linking of chemical or protein databases with WikiPathways.^16,17^

In this article, we show how using RDF makes the AOP-Wiki content more usable for automated exploration in combination with other existing semantic web-based information sources. We describe our implementation of Linked Open Data solutions for the AOP-Wiki to introduce new, effective ways of accessing and using the data. These solutions will enhance the usefulness of the AOP-Wiki to risk assessors, developers, and modelers, and facilitate answering complex research questions, also across databases or as part of automated workflows.

We hypothesize that with the implementation of RDF, with the use of standard ontologies for semantic modeling of information captured in AOPs, the data can be better exploited.^18^ Furthermore, the use of persistent, unique, and resolvable identifiers allows interoperability with other related data sources. When combined with computational tools that can access experimental data, these approaches can make AOP information a core element for predictive modeling.^19^

## Materials and Methods

### Registering AOP-Wiki identifiers in Identifiers.org

Before the development of the AOP-Wiki RDF, we registered the identifiers for the AOP, KE, KER, and stressor in the Minimum Information Required In the Annotation of Models (MIRIAM) Registry^20^ to allow Identifiers.org to resolve Internationalized Resource Identifiers (IRIs). To make all identifiers in the AOP-Wiki resolvable and linking to their corresponding database webpages, these IRIs, along with a variety of chemical and gene database identifier types, were implemented in the AOP-Wiki RDF.

### XML-to-RDF conversion code

The code for the XML-to-RDF conversion was written as a Jupyter notebook using Python version 3.7.3 in JupyterLab version 0.35.5, and is stored in GitHub (https://github.com/marvinm2/AOPWikiRDF).^21^

#### Downloading and parsing the AOP-Wiki XML

It downloads the AOP-Wiki XML quarterly download file of January 1, 2021 from https://aopwiki.org/downloads and parses the file with the ElementTree XML API Python library. Next, the Jupyter notebook stores all the AOP-Wiki content in a Python nested dictionary data model, one for each of the main components, which form the basis of the existing AOP-Wiki. These are the AOPs, KEs, KERs, stressors, chemicals, taxonomy, cell-terms, organ-terms, and the KE components, which comprise Biological Processes, Biological Objects, and Biological Actions.

#### Semantic annotation in the RDF

Terms from common biomedical terminologies and standard metadata vocabularies were used as predicates. These terms were retrieved from BioPortal^22^ or in the corresponding Web Ontology Language (OWL)^23^ files stored in GitHub. These ontologies include Dublin Core,^24^ DCMI Metadata Terms,^25^ RDF Schema,^26^ Friend Of A Friend,^27^ Adverse Outcome Pathway Ontology,^28^ Phenotypic Quality Ontology,^29^ Chemical Information ontology,^30^ NCI Thesaurus,^31^ Measurement Method Ontology,^32^ Simple Knowledge Organization System,^33^ National Center for Biotechnology Information Organismal Classification,^10^ Gene Ontology,^34^ EDAM bioinformatics operations, types of data, data formats, identifiers, and topics,^35^ Provenance, Authoring and Versioning,^36^ Vocabulary of Interlinked Datasets,^37^ Data Catalog Vocabulary.^38^

Table 1 provides an overview of these, including their prefixes and IRI patterns. Furthermore, the IRIs were completed for annotations that already exist in the AOP-Wiki such as the KE components, cell-terms, and organ-terms. These annotations include terms of the Cell Ontology,^39^ Uber-anatomy ontology,^40^ Gene Ontology,^34^ Molecular Interactions Controlled Vocabulary,^41^ Mammalian Phenotype Ontology,^42^ Medical Subject Headings,^43^ Human Phenotype Ontology,^44^ Population and Community Ontology,^45,46^ Neuro Behavior Ontology,^47^ Vertebrate trait ontology,^48^ PRotein Ontology,^49^ Chemical Entities of Biological Interest,^50^ and Foundational Model of Anatomy Ontology.^51^ These ontologies are listed in Supplementary Table S1 (Annex) together with their prefixes and IRI patterns.

**Table 1.** Ontologies and Vocabularies Used in the Resource Description Framework

Ontology name	Prefix in RDF	Base IRI
Dublin Core^24^	dc	http://purl.org/dc/elements/1.1/
DCMI Metadata Terms^25^	dcterms	http://purl.org/dc/terms/
RDF Schema^26^	rdfs	www.w3.org/2000/01/rdf-schema#
Friend Of A Friend^27^	foaf	http://xmlns.com/foaf/0.1/
Adverse Outcome Pathway Ontology^28^	aopo	http://aopkb.org/aop_ontology#
Phenotypic Quality Ontology^29^	pato	http://purl.obolibrary.org/obo/PATO_
Chemical Information Ontology^30^	cheminf	http://semanticscience.org/resource/CHEMINF_
NCI Thesaurus^31^	nci	http://ncicb.nci.nih.gov/xml/owl/EVS/Thesaurus.owl#
Measurement Method Ontology^32^	mmo	http://purl.obolibrary.org/obo/MMO_
Simple Knowledge Organization System^33^	skos	www.w3.org/2004/02/skos/core#
National Center for Biotechnology Information Organismal Classification^10^	ncbitaxon	http://purl.bioontology.org/ontology/NCBITAXON/
Gene Ontology^34^	go	http://purl.obolibrary.org/obo/GO_
EDAM bioinformatics operations, types of data, data formats, identifiers, and topics^35^	edam	http://edamontology.org/
Provenance, Authoring, and Versioning^36^	pav	http://purl.org/pav/
Vocabulary of Interlinked Datasets^37^	void	http://rdfs.org/ns/void#
Data Catalog Vocabulary^38^	dcat	http://www.w3.org/ns/dcat#

IRIs, Internationalized Resource Identifiers.

#### Addition of gene and protein identifiers

To increase the number of annotations and add more types of gene and protein identifiers for improved linking of data and repositories, the XML-to-RDF conversion includes two methods of mapping to gene and protein identifiers.

The first of which is based on existing Biological Object annotations with PRotein ontology (PR) terms^49^ in the AOP-Wiki, which were mapped to identifiers from NCBI Gene^52^ and UniProt,^53^ and symbols from the HUGO Gene Nomenclature Committee (HGNC)^54^ with the PR mapping file, promapping.txt, downloaded from https://proconsortium.org/download/current/ on May 10, 2020.

The second method involved textual gene identifier mapping for KEs and KERs, for which we extracted approved symbols, names, and synonyms for all human genes from the HGNC (downloaded from https://www.genenames.org/^54^ in January 2020). After loading the HGNC file, a symbol dictionary was created, which included all official gene symbols, names, and alternative gene names, and the library has been extended with variants of textual separators surrounding the symbols to avoid partial word overlaps. Next, text matching was done for each KE and KER description, the Molecular Initiating Event (MIE) and AO-specific section of KEs, and biological plausibility and empirical support sections of KERs. For each perfect match, the matching HGNC identifier was added to the KE and KER dictionaries to store KE-gene information.

#### BridgeDb identifier mapping

On top of the chemicals already present in the AOP-Wiki and the genes and proteins IDs added to the RDF with the textual mapping of HGNC symbols, we extended the coverage of external molecular databases using BridgeDb, an identifier mapping service for chemicals, genes, proteins, and interactions.^55^

The “requests” Python library (version 2.22.0) was used for calling BridgeDb's “xref” function to perform identifier mapping for chemicals and HGNC IDs that resulted from the textual mapping. The BridgeDb service was loaded with the Metabolite BridgeDb ID Mapping Database (version HMDB4.0.20190116-CHEBI193-WIKIDATA20201104, released on November 4, 2020)^56^ and the Gene/Protein BridgeDb ID Mapping Database (version 91, released on May 9, 2018).^57^ For chemicals, the CAS IDs from the AOP-Wiki XML were used as input to retrieve identifiers from ChEBI,^50^ ChemSpider,^58^ Wikidata,^59,60^ ChEMBL,^61^ PubChem,^62^ Drugbank,^63^ KEGG,^64^ LIPID MAPS,^65^ and HMDB.^66^ For genes, the HGNC IDs were used to request matching identifiers for NCBI Gene, UniProt, and Ensembl.^67^

#### File creation

**FIG. 1.**
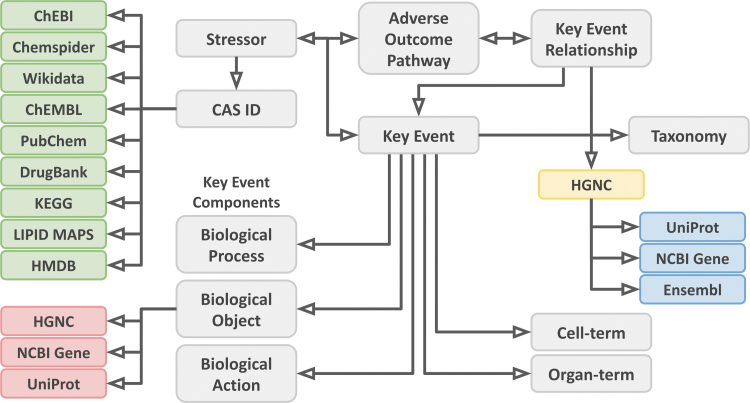
General overview of the AOP-Wiki RDF scheme. Arrows show the directional relationships described in the RDF. Gray boxes represent the basic elements of the AOP-Wiki. Green boxes indicate added chemical IDs using BridgeDb. Red boxes indicate added gene/protein IDs using Protein Ontology mapping. The yellow box indicates the text-mapped gene IDs, and the blue boxes indicate the added gene/protein IDs mapped from the text-mapped gene IDs using BridgeDb. RDF, resource description framework. Color images are available online.

All AOP-Wiki content, persistent identifiers, ontology annotations, and additional information for chemicals, genes, and proteins were stored into three RDF files using Turtle (ttl) syntax. While the central AOP-Wiki RDF file (AOP-Wiki.ttl) contains all existing AOP-Wiki components plus added chemical identifiers and identifiers mapped from PR terms in Biological Objects, the second file (AOP-Wiki-genes.ttl) contains all the text-mapped gene IDs and matching identifiers added by BridgeDb (Fig. 1). These files are accompanied by a metadata file (AOP-Wiki-void.ttl), which describes the datasets, code, and provenance, using standard vocabularies for semantic annotations of metadata, such as Dublin Core,^24,25^ Data Catalog Vocabulary,^38^ Friend of a Friend,^27^ and Vocabulary of Interlinked Datasets.^37^

### Validation and testing

#### Validation of the RDF

The RDF files were validated with the IDLab Turtle validator,^68^ an open-source RDF validator for Turtle syntax and XSD datatype errors.

#### Loading and testing the RDF

**FIG. 2.**
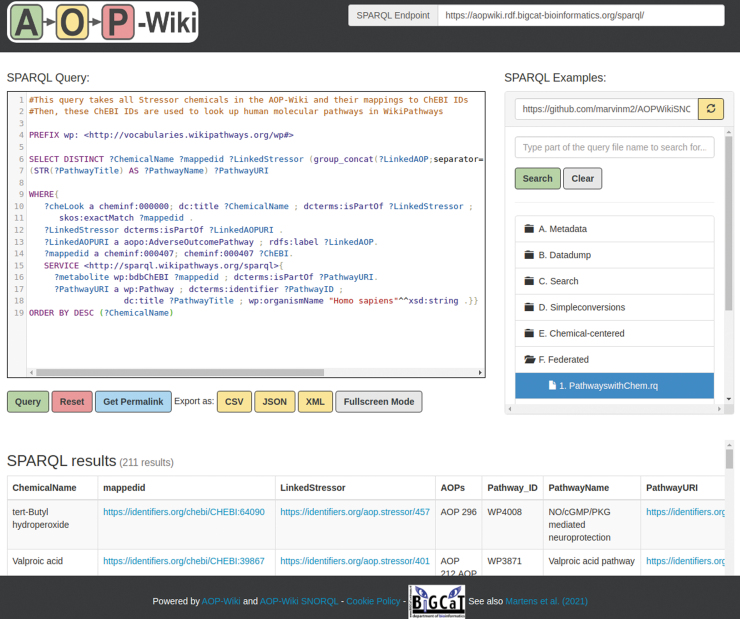
The AOP-Wiki SNORQL user interface. The AOP-Wiki SNORQL user interface allows for user-friendly access to the AOP-Wiki RDF by syntax highlighting and through providing a SPARQL examples panel (right panel). Color images are available online.

After validation of the RDF, the AOP-Wiki RDF was loaded in a public SPARQL endpoint (https://aopwiki.rdf.bigcat-bioinformatics.org/sparql) and is accessible through the developed SNORQL User Interface (https://aopwiki.rdf.bigcat-bioinformatics.org/, Fig. 2).

The data were tested with a Jupyter notebook that executes SPARQL queries through the SPARQL endpoint. These SPARQL queries retrieve metadata and the statistics for types of subjects, frequency of ontology usage, and the number of link-outs to the various databases.^21^ All SPARQL queries used for the testing of the RDF are available in the SPARQL examples panel in the AOP-Wiki SNORQL User Interface.

#### Validation of the SPARQL endpoint

On January 17, 2021, the AOP-Wiki SPARQL endpoint was registered in YummyData,^69^ which monitors compliance with Linked Data standards and scoring each SPARQL endpoint on availability, freshness, operation, usefulness, validity, and performance to calculate the Umaka Score. The scoring is done on a daily basis by performing SPARQL queries and HTTP requests related to the various measures for each aspect. YummyData also provides feedback on how to improve the score, which was used for improving the AOP-Wiki RDF and SPARQL endpoint.

## Results

The main result of this project is an RDF schema and scripts that lead to the production of RDF content for all AOP-Wiki content with additional semantic annotations, persistent identifiers, and extended identifiers for genes and chemicals, and consists of 122,576 unique triples consisting of 15,132 unique subjects, 158 unique predicates, and 53,087 unique objects (Fig. 1). The semantic annotation was done using 8 standard metadata vocabularies and 17 domain-specific ontologies and vocabularies. We here detail these results.

### Adverse outcome pathways

The 316 AOP subjects have 26 different types of predicates to create triples (Table 2). The overall most used vocabulary for predicates is Dublin Core and its extended set of terms, creating triples for the identifier, title, alternative title, creator, abstract, description, source, access rights, creation, and modification date for AOPs. The majority of these predicates also exist for other subjects. The AOP Ontology (AOPO) is used to connect AOPs to KE subjects with the predicates “has_key_event,” “has_molecular_initiating_event,” and “has_adverse_outcome,” and connect with KER subjects with the predicate “has_key_event_relationship.”

**Table 2.** Adverse Outcome Pathways and Their Properties in Resource Description Framework

Predicate	Object	Object example
Adverse outcome pathway		
a	aopo:AdverseOutcomePathway	
dc:identifier	Adverse Outcome Pathway (IRI)	aop:38
rdfs:label	Label (literal)	“AOP 38”
dc:title	Title (literal)	“Protein Alkylation leading to Liver Fibrosis”
dcterms:alternative	Alternative title (literal)	“Protein Alkylation to Liver Fibrosis”
dc:creator	Author (literal)	“““Brigitte Landesmann…
dcterms:abstract	Abstract (literal)	“““Hepatotoxicity in general is of special interest…
nci:C54571	Stressor (IRI)^*^	aop.stressor:9,aop.stressor: 13,aop.stressor:60,…
aopo:has_key_event	Key Event (IRI)^*^	aop.events:55,aop.events:1492,aop.events:1493, …
aopo:has_molecular_initiating_event	Key Event (IRI)^*^	aop.events:244
aopo:has_adverse_outcome	Key Event (IRI)^*^	aop.events:344
aopo:has_key_event_relationship	Key Event Relationship (IRI)^*^	aop.relationships:269,aop.relationships:1718, …
dc:description	Description (literal)	“““Two prototypical chemicals acting via protein alkylation are …
pato:0000047	Sex applicability (literal)	“Unspecific”
aopo:LifeStageContext	Life stage applicability (literal)	“Not Otherwise Specified”
aopo:AopContext	Applicability (literal)	“““The described AOP is valid for both sexes and any life stage …
edam:operation_3799	Quantitative considerations (literal)	“““More advanced in vitro models systems are needed …
aopo:has_evidence	Weight of evidence (literal)	“““Support for Essentiality of KEs …
nci:C25725	Potential applications (literal)	“““This systematic and coherent display of currently available …
nci:C25217	Overall assessment (literal)	“““Assessment of the Weight-of-Evidence supporting the AOP …
nci:C48192	Key Event essentiality (literal)	“““The essentiality of each of the KEs for this AOP …
dc:accessRights	AOP status (literal)	“Open for citation & comment”
foaf:page	Webpage (URL)	<https://identifiers.org/aop/38>
dcterms:created	Date of creation (literal)	“2016-11-29T18:41:16”
dcterms:modified	Date of latest modification (literal)	“2019-04-30T12:53:51”
dc:source	Source (literal)	“AOPWiki”

Asterisks indicate the object IRIs that connect to other subjects in the RDF.

Other terms of the AOPO were used for describing the overall applicability, life stage applicability, and weight of evidence. Furthermore, AOP subjects are unique to contain information on the quantitative considerations of the AOP, which is linked with “edam:operation 3799.” The link with stressors, overall assessment description, KE essentiality, and the potential applications of the AOP were annotated using the NCI Thesaurus terms “nci:C54571,” “nci:C25217,” “nci:C48192,” and “nci:C25725,” respectively. Finally, the sex applicability of AOPs is annotated with “pato:0000047,” which stands for biological sex.

### KEs and KERs

While the majority of triples for KEs and KERs have predicates identical to the ones for AOPs, there are properties that are unique to the 1131 KEs and 1363 KERs (Table 3). For KEs, these properties include measurement methods, level of biological organization, and structured information on cell-terms, organ-terms, Biological Processes, Objects, and Actions (Table 3). The measurement methods are coupled to 350 KEs with the predicate “mmo:0000000” from the Measurement Method Ontology, which stands for measurement method, and level of biological organization is linked with the “nci:C25664” to all KEs. Cell-terms and Organ-terms are described with the AOPO terms “aopo:CellTypeContext” and “aopo:OrganCotext,” respectively.

**Table 3.** Key Events and Their Properties in Resource Description Framework

Predicate	Object	Object example
Key event		
a	aopo:KeyEvent	
dc:identifier	Key Event (IRI)	aop.events:1502
rdfs:label	Label (literal)	“KE 1502”
dc:title	Title (literal)	“Histone deacetylase inhibition”
dcterms:alternative	Alternative title (literal)	“Histone deacetylase inhibition”
nci:C25664	Level of biological organization (literal)	“““Molecular”””
dc:description	Description (literal)	“““The inhibition of HDAC by HDIs is well conserved…
edam:data_1025	HGNC ID (IRI)^*^	hgnc:HDAC9,hgnc:MAA,hgnc:PRDX2
mmo:0000000	Measurement method (literal)	“““The measurement of HDAC inhibition monitors changes…
nci:C54571	Stressor (IRI)^*^	aop.stressor:340,aop.stressor:341,aop.stressor:342,…
aopo:CellTypeContext	Cell-term (IRI)^*^	cl:0000000
aopo:OrganContext	Organ-term (IRI)^*^	uberon:0000062
go:0008150	Biological process (IRI)^*^	go:0004857
pato:0001241	Biological object (IRI)^*^	pr:000008478
pato:0000001	Biological action (IRI)^*^	“WIKI:2”
ncbitaxon:131567	Taxonomy (IRI or literal)^*^	ncbitaxon:10116,”WCS_9606”,ncbitaxon:10090
pato:0000047	Sex applicability (literal)	“Unspecific”
aopo:LifeStageContext	Life stage applicability (literal)	“All life stages”
foaf:page	Webpage (URL)	<https://identifiers.org/aop.events/1502>
dcterms:isPartOf	Adverse Outcome Pathway (IRI)^*^	aop:212,aop:274,aop:275
dc:source	Source (literal)	“AOPWiki”
Key event relationship		
a	aopo:KeyEventRelationship	
dc:identifier	Key Event Relationship (URI)	aop.relationships:865
rdfs:label	Label (literal)	“KER 865”
aopo:has_upstream_key_event	Key Event (URI)^*^	aop.events:844
aopo:has_downstream_key_event	Key Event (URI)^*^	aop.events:845
dc:description	Description (literal)	“““One of the oxidation products of uroporphyrinogen…
nci:C80263	Biological plausibility (literal)	“““Reduced UROD enzyme activity, not protein levels…
edam:data_2042	Empirical support (literal)	“““Include consideration of temporal concordance…
nci:C71478	Uncertainties or inconsistencies (literal)	“““The precise mechanism of UROD inhibition has yet…
edam:data_1025	HGNC ID (IRI)^*^	hgnc:UROD
ncbitaxon:131567	Taxonomy (IRI or literal)^*^	ncbitaxon: 10090,ncbitaxon:10116,“WCS_9606”
pato:0000047	Sex applicability (literal)	“Unspecific”
aopo:LifeStageContext	Life stage applicability (literal)	“All life stages”,“Adult”,“Juvenile”
foaf:page	Webpage (URL)	<https://identifiers.org/aop.relationships/865>
dcterms:created	Date of creation (literal)	2016-11-29T18:41:35”
dcterms:modified	Date of latest modification (literal)	“2018-05-30T10:58:18”
dcterms:isPartOf	Adverse Outcome Pathway (URI)^*^	aop:131

Asterisks indicate the object IRIs that connect to other subjects in the RDF.

The Biological Process triples have the predicate “go:008150,” which stands for biological process, and the Biological Objects and Actions are linked with “pato:0001241” and “pato:0000001,” respectively. These ontological annotations are connected through IRIs of other subjects in the RDF. A shared predicate with AOPs is the term “nci:C54571” for MIEs that have links to stressors in the AOP-Wiki.

Properties that are specific to the 1363 KERs are the biological plausibility, empirical support, uncertainties, which we linked with the predicates “nci:C80263,” “edam:data_2042,” and “nci:71478.” These stand for the rationale, evidence, and uncertainty, respectively. Also, the RDF connects the upstream and downstream KEs of KERs with the terms “aopo:has_upstream_key_event” and “aopo:has_downstream_key_event” from the AOPO (Table 3).

### Stressors and chemicals

The RDF contains general stressor information such as descriptions and identifiers, and stressor triples describe their connections to MIEs and AOPs with “dcterms:isPartOf.” Sixty-three percent of the 523 stressors are also linked to chemicals with the predicate “aopo:has_chemical_entity” (Table 4).

**Table 4.** Stressors and Chemicals and Their Properties in Resource Description Framework

Predicate	Object	Object example
Stressor		
a	nci:C54571	
dc:identifier	Stressor (IRI)	aop.stressor:208
rdfs:label	Label (literal)	“Stressor 208”
dc:title	Title (literal)	“Gemfibrozil”
dc:description	Description (literal)	“““Fibrate drug”””
aopo:has_chemical_entity	Chemical identifier (IRI)^*^	cas:25812-30-0
foaf:page	Webpage (URL)	<https://identifiers.org/aop.stressor/208>
dcterms:created	Date of creation (literal)	“2016-11-29T18:42:27”
dcterms:modified	Date of latest modification (literal)	“2020-03-31T10:24:40”
dcterms:isPartOf	Adverse Outcome Pathway (IRI)^*^ Key Event (IRI)^*^	aop.events:227,aop.events:1170,aop:18,aop:37,aop:51,aop:61,aop:323, …
Chemical		
a	cheminf:000000	
	cheminf:000446	
dc:identifier	CAS identifier (IRI)	cas: 103-90-2
cheminf:000446	CAS identifier (literal)	“103-90-2”
dc:title	Title (literal)	“Acetaminophen”
dcterms:alternative	Synonyms (literal)	“4-Acetamidophenol”,“Paracetamol”, …
cheminf:000059	InChlKey (IRI)	inchikey:RZVAJINKPMORJF-UHFFFAOYSA-N
cheminf:000568	CompTox identifier (IRI)	comptox:DTXSID2020006
skos:exactMatch	Matched identifier (IRI)^*^	chebi:46195,chemspider:1906,wikidata:Q57055, …
dcterms:isPartOf	Stressor (IRI)^*^	aop.stressor:57

Asterisks indicate the object IRIs that connect to other subjects in the RDF.

The 329 chemical subjects are annotated with CAS and CompTox identifiers, and 320 also have InChIKeys, all of which we annotated with the Chemical Information Ontology (Table 4). Furthermore, the chemicals have predicate “skos:exactMatch” to link to all mapped chemical subjects present in the RDF, providing link-outs to nine additional external databases (Table 5). We annotated these with “rdf:type” and terms from the Chemical Information Ontology. In total, there are 3904 link-outs to 12 different chemical databases, allowing users to explore the AOP-Wiki by using their preferred type of chemical identifiers.

**Table 5.** Gene and Protein Databases, Their Type Annotation, and the Number of Identifiers Present in the Resource Description Framework

Database	rdfs:type	No. 1	No. 2
HGNC	edam:data_2298	97	846
Ensembl	edam:data_1033	—	813
Entrez Gene	edam:data_1027	32	804
UniProt	edam:data_2291	447	3653

Values in no. 1 are based on Protein Ontology mappings, and values in no. 2 are based on textual mapping with HGNC symbols.

HGNC, HUGO Gene Nomenclature Committee.

### Ontological annotations

Since the taxonomies, cell-terms, organ-terms, and the KE components all already have ontological annotations in the AOP-Wiki, they have the same properties that describe their type (Table 6), identifier, title, and source. These titles are based on the user-provided entries in the AOP-Wiki. Unique for the biological objects annotated with the PR is the inclusion of the “skos:exactMatch” predicate linking to 576 matching identifiers from UniProt, HGNC, and NCBI Gene (Table 7) to 126 PR tags, which are used in 166 KEs.

**Table 6.** Chemical Databases, Their Type Annotation, and the Number of Identifiers Present in the Resource Description Framework

Database	rdfs:type	No.
CAS	cheminf:000446	329
ChEBI	cheminf:000407	803
ChemSpider	cheminf:000405	343
ChEMBL compound	cheminf:000412	287
CompTox	cheminf:000568	329
Drugbank	cheminf:000406	161
HMDB	cheminf:000408	363
InChlKey	cheminf:000059	320
KEGG compound	cheminf:000409	264
Lipid maps	cheminf:000564	30
PubChem compound	cheminf:000140	338
Wikidata	cheminf:000567	328

**Table 7.** Cell-Terms, Organ-Terms, Taxonomies, Key Event Components, and Type Annotation in the Resource Description Framework

Subject	rdfs:type
Cell-term	aopo:CellTypeContext
Organ-term	aopo:OrganContext
Taxonomy	ncbitaxon:131567
Biological process	go:0008150
Biological object	pato:0001241
Biological action	pato:0000001

### Gene and protein identifiers

Extending the links of KEs and KERs with genes and proteins, RDF triples of 846 unique text-mapped gene identifiers on KEs and KERs are stored in a separate file. These make triples of KE and KER subjects link to the mapped HGNC identifiers with the predicate “edam:data_1025,” which stands for Gene identifier. These HGNC identifiers are subjects themselves, and have the “skos:exactMatch” predicate to link to matching identifiers from UniProt, NCBI Gene, and Ensembl, providing a total of 6001 link-outs using this method (Table 7).

### Federated SPARQL query example

The addition of external identifiers facilitates the execution of federated SPARQL queries to combine resources, such as the example shown in Figure 2, which is located in the SPARQL example panel under the folder “F. Federated.” The SPARQL query looks up all entities defined as a Chemical in the AOP-Wiki RDF with the predicate and subject “a cheminf:000000,” and extracts their names, mapped identifiers, and linked stressors. Next, the query looks for AOPs that mention the stressor. The next line in the query restricts the results by explicitly adding the type of “a aopo:AdverseOutcomePathway.” Similarly, the query makes sure that the mapped identifier of the chemical is from the ChEBI database by defining the mapped identifier as type “cheminf:000407.”

This is followed by the “SERVICE” statement, which defines the external SPARQL endpoint and defines the federated part of the query. In this example, the external SPARQL endpoint is WikiPathways (https://sparql.wikipathways.org/sparql), where the ChEBI identifier is used to match relevant pathways using “dcterms:isPartOf.” The last part of the SPARQL query retrieves information about the pathway and filters for human pathways. This SPARQL query results in a table of 211 rows with each chemical, their ChEBI identifier, related stressor and AOP, and the molecular pathways that the chemical is involved in. These linked pathways provide additional insights into the general functions of chemicals and their involvement in cellular processes and responses.

### Validation by YummyData

As an external validation of the developed SPARQL endpoint and RDF, YummyData indexes and ranks the AOP-Wiki SPARQL endpoint based on an array of tests on a daily basis (https://yummydata.org/endpoint/142). As of February 2021, the AOP-Wiki SPARQL endpoint is considered A rank with a Umaka score >80, consistently scoring above average on all aspects. Incidentally, the Umaka score drops slightly <80, giving it a B rank.

## Discussion

The work described in this article has led to the creation of AOP-Wiki RDF based on the existing AOP-Wiki XML, combined with a variety of ontologies and enriched with persistent identifiers. Besides, the data are extended with additional identifiers for chemicals, proteins, and genes. The RDF has been validated, loaded on a SPARQL endpoint, and tested using a Jupyter notebook, and is indexed in YummyData for external validation of the SPARQL endpoint. These developments made AOP-Wiki content ready for use in risk assessment workflows, through coding environments, or in federated SPARQL queries.

Because the AOPO is developed for consistent reporting in the domain of AOPs and allowing the integration of data and tools,^28^ it was an obvious choice to implement the AOPO for semantic annotations in the AOP-Wiki RDF. The ontology includes a variety of AOP-specific definitions for properties and classes, which are directly applicable to AOP-Wiki content in the RDF. However, these do not fully cover all types of entities and relationships that exist in the AOP-Wiki. For example, while it has terms to describe the connections between AOPs, KEs, and KERs, there is no annotation for the link with stressors. Similarly, terms are lacking for sex and taxonomic applicability, KE components, KER-specific information, and AOP assessment sections such as KE essentiality and quantitative considerations.

For the terms missing in the AOPO, we selected definitions from a wide range of other ontologies and vocabularies for the semantification of predicates and subjects, including NCI Thesaurus,^31^ NCBI of Organismal Classification, Gene Ontology, and Measurement Method Ontology. Whereas the majority of the AOP-Wiki contents are generic and can be described with well-established metadata ontologies, some properties of AOPs, KEs, and KERs could not be found, leading to the selection of more general terms, lacking detail that would be preferred. With the conversion to RDF, most of the necessary terms have been uncovered and documented, and these will be added to the AOPO.

Because the realm of AOPs includes many types of data, knowledge, repositories, and services, the development and implementation of a central, community-wide vocabulary would facilitate their integration. Since the AOPO has been developed to fill that purpose, it could be extended to include descriptions of classes and properties for all ontology terms used in the AOP-Wiki RDF. Having a central, field-wide ontology for AOPs helps maintain a high-quality vocabulary through continuous development and involvement of the community. Such an ontology would facilitate the annotation and integration of data, resources, and tools, as is done with the eNanoMapper ontology in the nanotoxicology community.^70^

With the increased importance of consistent use of identifiers to integrate knowledge and data, our implementation of persistent identifiers for AOPs, KEs, KERs, stressors, chemicals, proteins, and genes will benefit the integration of the AOP-Wiki with other resources, data, and tools.^71^ These persistent identifiers stored in the MIRIAM registry are stable, unique, resolvable, documented, and directly linked to the corresponding entries in the databases.^20,72^

Furthermore, our efforts have introduced additional content into the AOP-Wiki RDF through ID mappings and text-mapping for chemicals and genes, providing more ways of extrapolating the data and linking with other resources and data. While the addition of molecular descriptors of genes and proteins in KEs and KERs can provide more insights into the actual processes described, it is merely a small step toward complete machine-readable biological processes.

Although we have added molecular identifiers to increase the number of link-outs and improve the usefulness of the database, our addition of genes through textual ID mapping does introduce errors to the AOP-Wiki RDF. The automated process on free-text content assumes good practice in writing gene symbols and names according to the HGNC guidelines.^73^ Although HGNC strives for stable gene symbols and makes justified changes for problematic ones,^74^ some gene symbols still overlap with free-text abbreviations in the AOP-Wiki and are therefore falsely recognized.

Opportunities exist to improve the AOP-Wiki machine readability by having more structured text and annotations for molecular entities, pathways, organs, species, and other biological concepts that are relevant for AOPs and not yet covered by the KE components. Text-mining tools, such as ProMiner,^75^ ContentMine,^76^ and PolySearch2,^77^ could be implemented for extracting biological concepts and understanding associations to add more structured information to the AOP-Wiki, and facilitate the integration with other databases and tools. Once such concepts are recognized and extracted, the RDF could be extended and increase the interoperability of the AOP-Wiki with external databases, such as pathway databases.

The AOP-Wiki RDF allows for new and efficient ways of accessing the data and using it to answer questions. By loading the RDF in a SPARQL endpoint, SPARQL queries can be used to access the data and extract all necessary information. It allows complex queries across the complete AOP-Wiki database, optional filtering for any variable, and requesting an output suitable for answering the research question. Furthermore, these SPARQL queries can be executed from most coding environments as part of larger workflows or data pipelines.

It also facilitates direct linkage of databases through federated SPARQL queries, which returns information across databases with a single query. Any database with a SPARQL endpoint can be used for such questions across databases, such as WikiPathways,^16,17^ Wikidata,^59,60^ neXtProt,^15^ UniProt,^53^ ChEMBL-RDF,^78^ DisGeNET,^79,80^ Rhea,^81^ and Pathway Commons.^82^ Examples of federated queries are stored in the SPARQL examples panel in the AOP-Wiki SNORQL User Interface https://aopwiki.rdf.bigcat-bioinformatics.org/ (Fig. 2).

Another way of using the RDF to extract AOP-Wiki content is through a web service such as the git repository linked data API constructor (grlc),^83^ which can build a Web API on top of a SPARQL endpoint with predefined SPARQL queries. While more straightforward than SPARQL queries, the API is limited to the predefined SPARQL queries and variables implemented in these.

An advantage of creating RDF for the AOP-Wiki is the ability to link and expand AOP-Wiki content with information from other databases. For example, the Adverse Outcome Pathway Database combines knowledge from the AOP-Wiki with annotations of genes, chemicals, diseases, tissues, pathways, ontologies, and ToxCast data.^84,85^ Future work should focus on integrating such efforts by developing RDF, and thereby allow full integration of their data and tools^86^ with the AOP-Wiki RDF and other databases.

In terms of compliance with Linked Data standards according to the YummyData registry, the AOP-Wiki SPARQL endpoint consistently scores above average in availability, freshness, operation, usefulness, validity, and performance.^69^ With a consistent A rank, the AOP-Wiki SPARQL endpoint is placed among the 10% best scoring of the 70+ SPARQL endpoints registered in YummyData. However, incidentally the Umaka score dropped due to slow server response, or when the service has been down for maintenance or data loading. Based on the feedback given by YummyData, a point for improvement would be supporting more response formats of the SPARQL endpoint to increase the usefulness score.

## Conclusions

The implementation of compact identifiers and the development of a formal, machine-readable RDF schema make the content of the AOP-Wiki more findable and interoperable to other components of the database, and by allowing SPARQL queries and API to explore the data, the AOP-Wiki database was made accessible through new methods. Furthermore, the addition of link-outs to various chemical, gene, and protein databases, as well as the data storage in an RDF format and implementing Linked Open Data standards, has made the data more interoperable with other databases and tools.

Furthermore, the AOP-Wiki has recently introduced licenses on its content, and the code for the creation and validation of the AOP-Wiki RDF is available under the MIT license. These provide clear statements and terms of using, sharing, and modifying the content. Taken together with the addition of metadata and semantic information represented by ontology annotations, the content of the AOP-Wiki has been made more accessible and reusable. Therefore, the development of the AOP-Wiki RDF addresses all major FAIR principles.^13^

Overall, the AOP-Wiki RDF allows for new ways of exploring the data, using it in automated workflows, from coding environments, or directly through a SPARQL endpoint. With the implementation also comes the possibility to execute federated queries to combine data of multiple resources and answer more elaborate questions. For example, KEs can be linked to the results of ToxCast assays that measure the activity of the protein described, or molecular pathways can be explored for a more detailed description of mechanistic processes.

## Supplementary Material

Supplemental data
