# Supplementary material for: Providing Adverse Outcome Pathways from the AOP-Wiki in a Semantic Web Format to Increase Usability and Accessibility of the Content
Source: Appl In Vitro Toxicol. 2022 Mar 17;8(1):2–13. doi: 10.1089/aivt.2021.0010 (PMC8978481; doi:10.1089/aivt.2021.0010)
Supplement: Supplemental data [file Suppl_TableS1.docx]

**Table S1: Prefixes in the RDF for the Key Event Component annotations**

| **Ontology name** | **Prefix in RDF** | **Base IRI** |
| --- | --- | --- |
| Cell Ontology^39^ | cl | <http://purl.obolibrary.org/obo/CL_> |
| Uber-anatomy ontology^40^ | uberon | <http://purl.obolibrary.org/obo/UBERON_> |
| Gene Ontology^34^ | go | <http://purl.obolibrary.org/obo/GO_> |
| Molecular Interactions Controlled Vocabulary^41^ | mi | <http://purl.obolibrary.org/obo/MI_> |
| Mammalian Phenotype Ontology^42^ | mp | <http://purl.obolibrary.org/obo/MP_> |
| Medical Subject Headings^43^ | mesh | <http://purl.bioontology.org/ontology/MESH/> |
| Human Phenotype Ontology^44^ | hp | <http://purl.obolibrary.org/obo/HP_> |
| Population and Community Ontology^45,46^ | pco | <http://purl.obolibrary.org/obo/PCO_> |
| Neuro Behavior Ontology^47^ | nbo | <http://purl.obolibrary.org/obo/NBO_> |
| Vertebrate trait ontology^48^ | vt | <http://purl.obolibrary.org/obo/VT_> |
| PRotein Ontology^49^ | pr | <http://purl.obolibrary.org/obo/PR_> |
| Chemical Entities of Biological Interest^50^ | chebio | <http://purl.obolibrary.org/obo/CHEBI_> |
| Foundational Model of Anatomy Ontology^51^ | fma | <http://purl.org/sig/ont/fma/fma> |
